# Transcatheter edge-to-edge repair of tricuspid regurgitation in the Netherlands: state of the art and future perspectives

**DOI:** 10.1007/s12471-022-01673-z

**Published:** 2022-03-29

**Authors:** Jan A. Krikken, Ad F. M. van den Heuvel, H. Marco Willemsen, Adriaan A. Voors, Erik Lipsic

**Affiliations:** Department of Cardiology, University Medical Centre Groningen, University of Groningen, Groningen, The Netherlands

**Keywords:** Tricuspid valve regurgitation, Edge-to-edge repair, Transcatheter valve intervention, Heart failure

## Abstract

Despite the high prevalence and adverse clinical outcomes of severe tricuspid regurgitation (TR), conventional treatment options, surgical or pharmacological, are limited. Surgery is associated with a high peri-operative risk and medical treatment has not clearly resulted in clinical improvements. Therefore, there is a high unmet need to reduce morbidity and mortality in patients with severe TR. During recent years, several transcatheter solutions have been studied. This review focuses on the transcatheter edge-to-edge repair of TR (TTVR) with respect to patient selection, the procedure, pre- and peri-procedural echocardiographic assessments and clinical outcomes. Furthermore, we highlight the current status of TTVR in the Netherlands and provide data from our initial experience at the University Medical Centre Groningen.

## Introduction

Severe tricuspid regurgitation (TR) is a serious condition associated with high cardiovascular morbidity and mortality [1–3]. Nevertheless, surgical and pharmacological treatment options have been underexposed for years. Thanks to extensive developments in TR treatment in the last decade, the tricuspid valve (TV) can no longer be referred to as the ‘forgotten valve’.

Secondary, or functional, TR is most prevalent in our Western society and the condition is often linked to left-sided heart disease or atrial fibrillation [4]. Right ventricular (RV) dysfunction and progressive TV annular dilation play a role in this process and are not always stopped when the underlying condition is treated. Therefore, our current guidelines set a low threshold for concomitant TV repair when left-sided heart surgery is performed [5, 6].

In cases of isolated severe primary or secondary TR, TV surgery is recommended when patients are symptomatic or when RV dysfunction is present [6, 7]. However, peri-operative mortality for isolated TV surgery is high (up to 11%) and, although surgery may result in relief of symptoms, to date no significant difference in survival has been seen when compared to medical therapy [8, 9]. As a consequence, in elderly TR patients and those with previous cardiac surgery, the risks of surgery do not outweigh potential benefits and treatment is often limited to diuretics for symptom relief.

Driven by an emerging demand for minimally invasive treatment of severe TR, a wide variety of transcatheter techniques has been developed in recent years [10]. This review focuses on the most ‘mature’ and widely accepted procedure: edge-to-edge repair of the TV (TTVR). We discuss the evidence and scope of the therapy. Moreover, the important role of echocardiography in patient selection and intraprocedural guidance is evaluated along with the current status of the procedure in the Netherlands in general and Groningen in particular.

## Transcatheter treatment of severe TR: edge-to-edge repair

### Evidence

In the last decade we have experienced ongoing evolution in the field of transcatheter treatment of severe TR. Several new techniques and inventive applications of existing devices have been developed. Essentially, these techniques represent a transcatheter equivalent of the conventional surgical techniques for TV surgery: edge-to-edge repair (e.g. Mitraclip/Triclip (Abbott Vascular, Santa Clara, CA, USA); PASCAL (Edwards Lifesciences, Irvine, CA, USA)), different annuloplasty techniques (e.g. Cardioband (Edwards Lifesciences); Millipede (Millipede Inc., Santa Rosa, CA, USA); Trialign (Mitralign, Tewksbury, MA, USA)) or even complete percutaneous valve replacement (e.g. NaviGate (NaviGate Cardiac Structures Inc., Lake Forest, CA, USA); Tricento (NVT GmbH, Lotzenäcker, Germany)) [10].

Currently, most experience has been gained with transcatheter edge-to-edge repair (TTVR) using a technique similar to transcatheter repair of the mitral valve. Initially, numerous operators used the MitraClip system in the tricuspid position and more recently the TriClip system was introduced, which is essentially a modification of the MitraClip system [11–16]. In addition, increasing experience is being gained with the PASCAL TR system. This is another edge-to-edge device with a central spacer (for occupation of the regurgitant orifice) in addition to the approximation system. During the last 3 years, observational data from registries and prospective single-arm studies have shown promising results of percutaneous TV repair [14, 17–19], while there is no evidence for a difference in outcome between Mitraclip/Triclip and PASCAL TR [20]. These studies showed a consistent reduction in TR in the majority of cases. This reduction was accompanied by an improvement of symptoms and increase in quality of life, which proved to be durable over time [17]. Moreover, intervention reduced the annual rate of heart failure hospitalisations as compared to pre-procedure [21]. In a propensity-matched case-control study (TriValve registry) transcatheter TV intervention was associated with a lower 1‑year mortality and rehospitalisation rate as compared to matched controls [22]. Based on these early experiences, transcatheter treatment for symptomatic TR received a IIb (may be considered) recommendation for inoperable patients in the latest guidelines [6].

Importantly, no randomised controlled trials compared TTVR to (optimal) medical treatment. However, recently two randomised studies have started that will hopefully provide more decisive evidence for TTVR, as described in more detail later.

### Procedure in brief

In general, TTVR (TriClip and MitraClip in the TV position) is performed with the patient under general anaesthesia. Vascular access is via the femoral vein. During the entire procedure fluoroscopy and transoesophageal echocardiography (TEE) are used for visualisation of cardiac anatomy and the device delivery system. The delivery system (guide) and catheter are advanced into the right atrium until the clip emerges from the guide. Steering is done by the use of control knobs and levers as described earlier [15, 18], aiming for the site of maximal regurgitation and to achieve optimal clip position and a coaxial approach to the intended valve coaptation plane. Afterwards the TV is crossed and clip position and perpendicularity are evaluated by TEE. Clip grasping and residual TR are also assessed by TEE (see below for more details about intraoperative TEE guiding). As per the accepted standard operating procedures for MitraClip and TriClip use, fluoroscopy is used throughout the procedure to ensure appropriate gross directionality of the equipment and to visualise clip opening and closing during grasp preparation. After placement of an initial clip, typically in the anteroseptal commissure, a second and/or a third clip can be placed if desired. Each clip is delivered following the above-mentioned steps.

### Patient selection

Current ESC guidelines recommend conventional surgical TV repair in cases of severe TR with symptoms and/or signs of heart failure and RV dysfunction [6, 7]. However, as already stated, the surgical risk involved in an isolated TV intervention is often high. This is probably not merely a pure surgical phenomenon but also reflects the frail condition of these patients. In light of this, it is illustrative to note the mean EuroSCORE II in the TriValve registry and TRILUMINATE trial, which was 12 ± 11% and 8.7 ± 11%, respectively [18, 19]. Therefore, a thorough ‘heart team’ discussion is warranted before a treatment strategy is chosen. When a patient is declined for surgery due to a high estimated surgical risk, the current literature suggests that TTVR is a promising alternative in addition to diuretic treatment.

TR severity is an independent predictor of mortality and morbidity in the outpatient clinic population with the worst prognosis in patients with grade 4 and 5 TR as compared to grade 3 (as reflected in the ‘new’ classification system, see below) [23, 24]. Furthermore, TTVR failure or a large residual TR is a predictor of mortality and morbidity [14]. Failure of TTVR is mostly determined by TR severity and worse RV function as reflected by a greater effective regurgitant orifice area (EROA), larger coaptation gap and a greater tenting area. Furthermore, a non-central or non-anteroseptal maximum jet location is associated with TTVR failure [14, 25]. This might reflect the greater technical challenge in non-central or non-anteroseptal TR. However, the underlying TR mechanism might also be of influence, since a secondary TR, in general, would not cause such a jet.

In patients with severe TR, pulmonary hypertension (PH) is a prevalent condition that may even be involved in the pathophysiological basis of TR development and deterioration. PH in association with left-sided heart failure (NICE group 2) is by far the most prevalent form of PH, accounting for 50–85% of cases [26]. It therefore seems reasonable to pursue an optimal left-sided therapy before initiating a TV intervention, be it by treating significant aortic or mitral valve disease [27, 28] or by optimising medical therapy for (left-sided) heart failure [29]. Although the presence of PH had no significant impact on outcome in the TriValve registry, the TRILUMINATE trial excluded patients with a systolic pulmonary artery pressure > 60 mm Hg and (surgical) treatment is discouraged in these patients [7, 18]. Our current knowledge therefore warrants a reticent approach in cases of severe TR in the presence of PH, especially when RV dysfunction is present and TR may function as an ‘escape’ valve in a compromised pressure-loaded right ventricle.

A considerable proportion (14–24%) of TR patients may have a pacemaker or implantable cardioverter defibrillator (ICD) lead crossing the TV [17]. However, TR is considered to be ‘lead-induced’ in only a minority of these patients. Presence of a lead as such should not be considered to be a contraindication for TV clipping. However, the lead may impede proper intraprocedural echocardiographic visualisation or interfere with clip positioning.

Tab. 1 offers a summary of recommendations for patient referral and selection. Of note is that patient selection requires an integrated approach with patient and echocardiographic characteristics taken into account. Absolute cut-offs regarding, for instance, coaptation gap and EROA are difficult to determine and may change with increased experience and device development. For example, earlier recommendations regarded a coaptation gap > 7 mm to be least suitable for TTVR [30]. However, recent experience showed that even when the coaptation gap is > 10 mm treatment success can be achieved [31].

**Table 1:** Recommended criteria for deciding which patients should be selected for transcatheter edge-to-edge tricuspid valve repair

Symptomatic, severe TR (> NYHA I)
Life expectancy more than 1 year
High surgical risk and declined for conventional surgery as determined by ‘heart team’
Optimal treatment of left-sided heart failure and/or valvular disease
Absence of (severe) pulmonary hypertension (SPAP < 60 mm Hg, estimated by echocardiography)
TR classified as ‘secondary’ (annulus dilation > 40 mm)
Optimal visualisation by TEE; pre-procedural screening!
Central or anteroseptal jet location of TR
Absence of PM/ICD lead or PM/ICD lead not involved in main TR mechanism or impairing visualisation or grasping
Normal or maximum moderately reduced right ventricular function

*TR* tricuspid regurgitation, *SPAP* systolic pulmonary arterial pressure, *TEE* transoesophageal echocardiography, *PM* pacemaker, *ICD* implantable cardioverter defibrillator

### Safety

The current literature reports a relatively low adverse event rate following TTVR, especially when considering the frail condition of these patients. At 1 year, major adverse events were reported in 7% of patients. One-year mortality rates were between 7% and 19% [14, 17].

Bleeding complications seem the most important event directly related to the intervention and can occur in up to ~ 11% of cases [17]. This is important, as access bleeding may impact prognosis [32]. Therefore, this issue needs further attention in future studies. Single leaflet device attachment was reported in 5 of 84 cases in the TRILUMINATE trial. This had no impact on TR severity. Embolisation of a clip was not reported in this study.

## Echocardiography

### Grading of TR severity

Current guidelines recommend a ‘3-step’ system for grading TR severity. This grading system takes both qualitative and quantitative parameters into account [33]. In clinical practice, however, grading is still largely based on qualitative measures alone. In general, TR is graded as ‘severe’ when the vena contracta is ≥ 7 mm and the EROA is ≥ 40 mm^2^. Remarkably, a considerable proportion of TR patients are encountered with a wide range of TR severity who all fall within the ‘severe’ grade according to the current guidelines. A percutaneous intervention that reduces TR from, for instance, an EROA of 82 mm^2^ to 45 mm^2^ will qualify for a reduction from ‘severe TR’ to ‘severe TR’ in the old system, despite a huge reduction in TR. Therefore, a new grading system was recently proposed, refining the TR definition by expanding the ‘severe’ grade through the addition of a grade 4 (‘massive’) and grade 5 (‘torrential’) to the existing system (Tab. 2; [34]). Apart from representing a refinement, this new classification has also had an impact on cardiovascular prognosis, with worse outcomes in patients with grade 4 and 5 TR [23, 24]. In line with this is the observation that if TR after TTVR is still grade 3 (‘severe’) this does not necessarily reflect a procedural failure. A considerable proportion of patients in the TRILUMINATE trial (42% after 6 months, *n* = 70; 30% after 1 year, *n* = 63) and TriValve registry have a grade 3 residual TR but with a reduction of severity from grade 4 or 5 [17, 18]. This reduction might still have an impact on symptoms and functional capacity [35]. Obviously, this hypothesis needs further confirmation in prospective trials.

**Table 2:** New 5‑step classification system for tricuspid regurgitation [34]

Variable	1: ‘Mild’	2: ‘Moderate’	3: ‘Severe’	4: ‘Massive’	5: ‘Torrential’
VC	< 3 mm	3–6.9 mm	7–13 mm	14–20 mm	≥ 21 mm
EROA	< 20 mm^2^	20–39 mm^2^	40–59 mm^2^	60–79 mm^2^	≥ 80 mm^2^

*VC* vena contracta, *EROA* effective regurgitant orifice area

### Echocardiographic evaluation and intraoperative echocardiographic guidance

Echocardiography is the most important imaging modality for determining TR severity and aetiology. Also, when planning a TTVR, vigorous pre-procedural evaluation by both transthoracic echocardiography (TTE) and TEE is paramount for the success of the procedure. TTE is essential for quantifying TR severity. Furthermore, TTE will reveal the aetiology of TR in the majority of cases and give further insights into RV and LV function and concomitant left-sided valve dysfunctions.

In addition to TTE, we recommend that TEE be performed in the clipping centre, prior to TTVR, to evaluate visualisation of the TV in the different ‘standard views’ (transgastric short axis and RV inflow and outflow view; see next paragraph) and to ensure optimal guiding during the procedure. Of note is that in contrast to mitral valve clipping procedures, where 3D imaging is indispensable to ensure perpendicularity of the clip, 3D visualisation of the TV is more difficult and the quality is often insufficient. Therefore, the transgastric short axis view is generally used for this purpose. In addition to imaging quality, TEE will give further insights into the TR aetiology and the location of maximum regurgitation. Moreover, in the presence of a pacemaker or ICD lead, TEE will indicate: (1) whether the lead is involved in the TR aetiology, (2) the lead location and whether this is likely to hamper clip positioning and adequate visualisation.

**Fig. 1:**
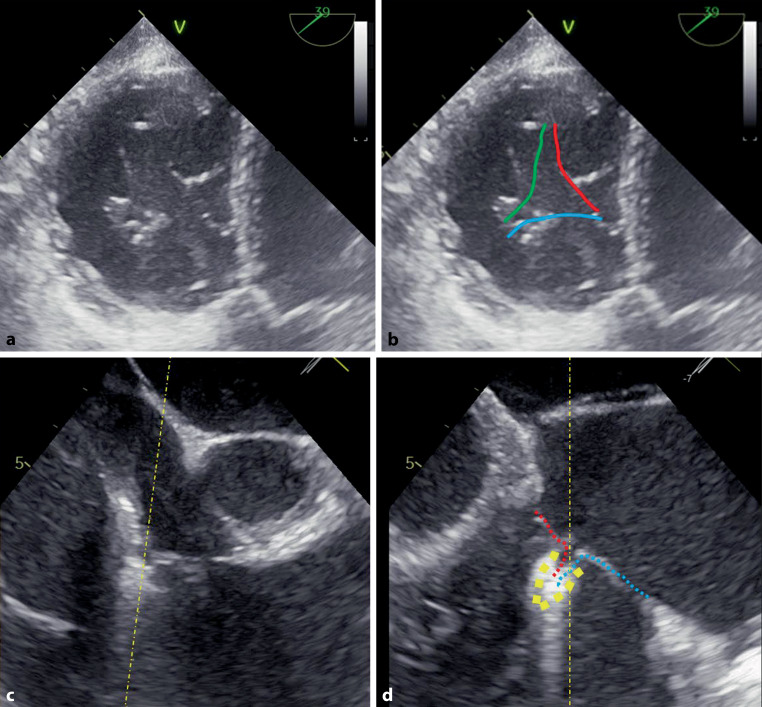
**a**–**c** Visualisation of tricuspid valve clipping by transoesophageal echocardiography. **a** Transgastric short axis view of the tricuspid valve. **b** Same view as in (**a**); the three leaflets are highlighted: *red* septal, *green* posterior, *blue* anterior. **c and d** Cross-plane image in mid-/deep oesophageal position showing the septal (*red*) and anterior (*blue*) leaflet positioned in a closed clip (*yellow*)

During the TTVR procedure, positioning of the delivery system is guided by TEE in addition to fluoroscopy. For procedural success, as for the MitraClip, positioning of the clip perpendicular to the line of coaptation of the TV leaflets is paramount. The transgastric short axis view (usually 20° to 50°) is the only two-dimensional TEE view in which all three leaflets can be evaluated simultaneously and in which perpendicularity can be evaluated adequately (Fig. 1). Grasping is usually assessed in the mid-/deep oesophageal position with the use of cross-plane imaging (Fig. 1). Finally, TEE is used for evaluation of leaflet insertion and residual TR. Of note is that the maximum regurgitation jet is most prevalent in the central or anteroseptal position. Consequently, the anteroseptal or posteroseptal commissure is generally targeted for clipping. This may also reduce annular dimensions and promote favourable remodelling. Furthermore, placing the first clip in the anteroseptal position is unlikely to hamper echocardiographic visualisation when placing a second clip. Clipping the anterior and posterior leaflets is generally not advised because this may distort the valve and worsen TR [36].

## Edge-to-edge repair in the Netherlands: the Groningen experience

In the wake of international developments, the importance of transcatheter repair for severe TR is increasingly being recognised in the Netherlands. Currently, TTVR is performed in five Dutch medical centres: Amsterdam University Medical Centre, Erasmus Medical Centre Rotterdam, St. Antonius Hospital Nieuwegein, Leiden University Medical Centre and the University Medical Centre Groningen. As in other countries the initial experiences were with the off-label use of the conventional MitraClip system in the TV position. In 2016, we were the first to report on a percutaneous TV repair using MitraClip for the treatment of severe TR in a patient with congenitally corrected transposition of the great arteries [37]. Last year the TriClip system, which is essentially a modification of the MitraClip system, was introduced in the Netherlands. To date, around 30 ‘off-label’ MitraClip TV repairs and 60 TriClip procedures have been performed in the Netherlands.

In our centre, we gained a reasonable amount of experience by performing a total of 17 TV clipping procedures. Starting in 2018, the first ten patients were treated with the conventional MitraClip system. As of the autumn of 2020 the dedicated TriClip system was introduced at our centre. Since then seven consecutive patients have been treated with TriClip.

Tab. 3 shows the characteristics of the patients treated in our centre. Except for one patient with brain metastasis, all patients were declined for conventional surgery because of previous cardiac surgery and/or frailty. Mean age was 78 years with a range between 52 and 87. Mean EuroSCORE II was relatively high with an average value of 7.1 ± 5.7%. Fifteen out of the 17 patients had a history of atrial fibrillation. Ten patients had a history of cardiothoracic surgery. Serum creatinine and N‑terminal pro-brain natriuretic peptide levels were moderately elevated and RV function was more or less impaired (tricuspid annular plane systolic excursion (TAPSE) < 17 mm and/or tricuspid annular systolic velocity (S’RV) < 9.5 cm/s) in 11 patients. In 15 patients successful clip delivery was achieved. In one patient intraprocedural imaging was unexpectedly disappointing, probably because of interference with the delivery system; hence the procedure was aborted. In another patient clip delivery was not achieved due to a very prominent eustachian valve which obliterated sufficient access to the right atrium and TV. A total of 31 clips were delivered in the remaining 15 patients (1–3 per patient). The anteroseptal commissure was targeted for 29 of the clips. Two clips were delivered in the posteroseptal commissure.

**Table 3:** Characteristics of patients treated with transcatheter edge-to-edge tricuspid valve repair at the University Medical Centre Groningen

Males/females	6/11
*MitraClip*^*a*^*/TriClip*	10/7
*Age (median, range)*	78 (52–87) years
*EuroSCORE II (mean* *±* *SD)*	7.1 ± 5.7%
*Prior open heart surgery*:	10 (59%)
CABG	4
Aortic valve replacement	2
Mitral valve repair/replacement	5
Other	1
*Prior percutaneous mitral valve repair (MitraClip)*	3
*Prior TAVR*	1
*Atrial fibrillation*	15 (88%)
*Diabetes*	3 (18%)
*Creatinine (mean* *±* *SD)*	1.06 (0.34) mg/dl
*NT-proBNP (median, range)*	1031 (102–7205) pg/ml
*Left ventricular ejection fraction (mean* *±* *SD)*	46 ± 11 (%)
*TAPSE (mean* *±* *SD)*	16.8 ± 4.7 mm
*S’RV*	9.0 ± 2.5 cm/s

*EuroSCORE* European System for Cardiac Operative Risk Evaluation, *CABG* coronary artery bypass grafting, *TAVR* transcatheter aortic valve replacement, *SD* standard deviation, *NT-proBNP* N-terminal pro-brain natriuretic peptide, *TAPSE* tricuspid annular plane systolic excursion, *S’RV* tricuspid annular systolic velocity

^a^MitraClip in tricuspid valve position

**Fig. 2:**
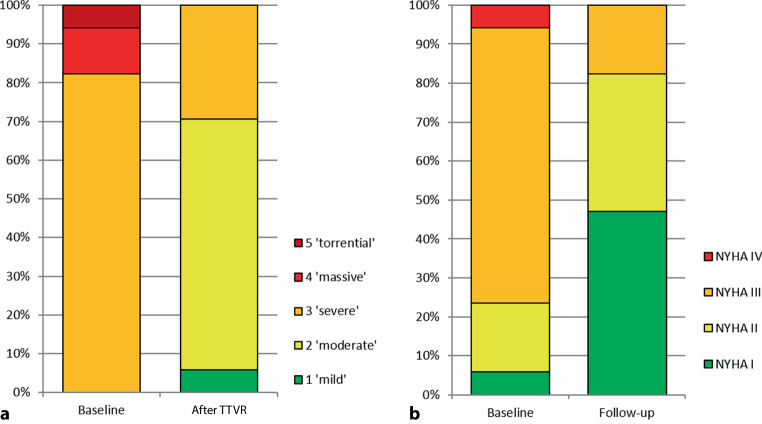
**a** Tricuspid regurgitation severity before and after transcatheter edge-to-edge tricuspid valve repair (*TTVR*). **b** New York Heart Association (*NYHA*) functional class at baseline and follow-up

At baseline, all patients had a grade 3 TR or worse. Two patients were classified as grade 4 and 1 patient had grade 5 TR according to the new classification system. After TTVR, TR was reduced to grade 2 or less in 70% of the patients. Five patients still had a severe TR after TTVR. Obviously, these include the two above-mentioned patients in whom clip delivery was not successful. The remaining three patients had a pre-procedural grade 4 or 5 TR, which was reduced but still severe (Fig. 2a). The reduction of TR was accompanied by an improvement of symptoms (NYHA class) during follow-up as visualised in Fig. 2b. At baseline 76% of patients had NYHA class 3–4, which was reduced to 18% during follow-up (mean 41 days). Long-term follow-up data were available for only some of the patients, as many procedures were performed in late 2020 and early 2021, but in general NYHA class and TR class were consistent with the short-term findings.

Only one serious peri-procedural complication occurred when a patient had a transient third-degree atrioventricular block due to catheter manipulation. The patient had to be resuscitated and temporary transvenous pacing was performed. There were no clinically significant bleedings. There was one single leaflet detachment which could be adequately treated with an additional clip. There were no cases of significant TV stenosis.

In the first 30 days after the procedure there were no hospital readmissions. Unfortunately one patient, in whom the clipping was unsuccessful, died within this period due to progressive end-stage heart failure. During follow-up two more patients died because of progressive heart failure (146 and 335 days after TTVR respectively).

In conclusion, our data show that in our ‘real-life programme’ TTVR in patients not amenable to surgery, is feasible, safe and leads to a reduction of TR and accompanying symptoms in the majority of patients. Patient characteristics are generally comparable to those of other study populations, as are our findings with respect to TR reduction and NYHA class.

## Future perspectives

Due to the significant morbidity and mortality associated with severe TR and the high risk of conventional surgery, additional options for transcatheter TV intervention have been developed in recent years. Of these different techniques, edge-to-edge repair by clipping (TTVR) is considered the most mature; hence the majority of available evidence comes from this field. The technique is generally considered safe and feasible and available non-randomised data suggest a consistent benefit with regard to symptoms, functional status, hospitalisations and survival. Consequently, transcatheter treatment of severe TR was given a IIb (may be considered) recommendation in the youngest ESC guidelines on valvular disease [6]. Interestingly, the recently started TRILUMINATE pivotal trial (NCT03904147) and CLASP II TR trial (NCT04097145) make a direct comparison between optimal medical treatment and TriClip and PASCAL devices, respectively. These first randomised trials will evaluate a total of 1525 patients, both having (hierarchical) composite primary endpoints containing all-cause mortality, heart failure admissions and quality of life. These ongoing studies along with the emerging developments in other tricuspid-targeting percutaneous (transcatheter) techniques are abruptly changing our view of how to treat severe TR. This may prelude a higher appraisal of TTVR in future guidelines in line with the recent advances in mitral valve interventions.

The future developments will reveal whether this new hope is justified. Currently, an increasing number of operators are gaining experience with the TriClip system and the per-centre and per-operator numbers are increasing steadily. Unfortunately, in the Netherlands TTVR is currently not reimbursed, hindering the opportunities for expanding our experience and increasing the number of procedures. Hence, we are clearly in danger of missing the boat. Therefore, the current efforts of the ‘TTVR community’ in the Netherlands aim at initiating a randomised trial focusing on the efficacy and cost-effectiveness of TV clipping in the specific Dutch situation. TV clipping is a promising new therapy, an opportunity we cannot afford to let pass.
